# Effects of mineral methionine hydroxy analog chelate in sow diets on epigenetic modification and growth of progeny

**DOI:** 10.1093/jas/skaa271

**Published:** 2020-08-25

**Authors:** Ki Beom Jang, Jong Hyuk Kim, Jerry M Purvis, Juxing Chen, Ping Ren, Mercedes Vazquez-Anon, Sung Woo Kim

**Affiliations:** 1 Department of Animal Science, North Carolina State University, Raleigh, NC; 2 N.G. Purvis Farm Inc., Robbins, NC; 3 Novus International, Inc., St. Charles, MO

**Keywords:** chelated minerals, growth, histone acetylation, intestinal health, piglets, sows

## Abstract

The study was conducted to determine the effects of mineral methionine hydroxy analog chelate (**MMHAC**) partially replacing inorganic trace minerals in sow diets on epigenetic and transcriptional changes in the muscle and jejunum of progeny. The MMHAC is zinc (Zn), manganese (Mn), and copper (Cu) chelated with methionine hydroxy analog (Zn-, Mn-, and Cu-methionine hydroxy analog chelate [**MHAC**]). On day 35 of gestation, 60 pregnant sows were allotted to two dietary treatments in a randomized completed block design using parity as a block: 1) **ITM**: inorganic trace minerals with zinc sulfate (ZnSO_4_), manganese oxide (MnO), and copper sulfate (CuSO_4_) and 2) **CTM**: 50% of ITM was replaced with MMHAC (MINTREX trace minerals, Novus International Inc., St Charles, MO). Gestation and lactation diets were formulated to meet or exceed NRC requirements. On days 1 and 18 of lactation, milk samples from 16 sows per treatment were collected to measure immunoglobulins (immunoglobulin G, immunoglobulin A, and immunoglobulin M) and micromineral concentrations. Two pigs per litter were selected to collect blood to measure the concentration of immunoglobulins in the serum, and then euthanized to collect jejunal mucosa, jejunum tissues, and *longissimus* muscle to measure global deoxyribonucleic acid methylation, histone acetylation, cytokines, and jejunal histomorphology at birth and day 18 of lactation. Data were analyzed using Proc MIXED of SAS. Supplementation of MMHAC tended to decrease (*P* = 0.059) body weight (**BW**) loss of sows during lactation and tended to increase (*P* = 0.098) piglet BW on day 18 of lactation. Supplementation of MMHAC increased (*P* < 0.05) global histone acetylation and tended to decrease myogenic regulatory factor 4 messenger ribonucleic acid (**mRNA**; *P* = 0.068) and delta 4-desaturase sphingolipid1 (**DEGS1**) mRNA (*P* = 0.086) in *longissimus* muscle of piglets at birth. Supplementation of MMHAC decreased (*P* < 0.05) nuclear factor kappa B mRNA in the jejunum and *DEGS1* mRNA in *longissimus* muscle and tended to decrease mucin-2 (**MUC2**) mRNA (*P* = 0.057) and transforming growth factor-beta 1 (**TGF-β1**) mRNA (*P* = 0.057) in the jejunum of piglets on day 18 of lactation. There were, however, no changes in the amounts of tumor necrosis factor-alpha, interleukin-8, TGF-β, MUC2, and myogenic factor 6 in the tissues by MMHAC. In conclusion, maternal supplementation of MMHAC could contribute to histone acetylation and programming in the fetus, which potentially regulates intestinal health and skeletal muscle development of piglets at birth and weaning, possibly leading to enhanced growth of their piglets.

## Introduction

With intensive genetic selection for the prolificacy of sows, the swine industry is challenged with improving the piglet survival rate in connection with the increased litter size and decreased litter uniformity (Kim and Hansen, 2013). It is well known that maternal nutrition can have an influence on not only the development of fetal organ and tissue but also milk yield (Kim et al., 1999; McPherson et al., 2004; Farmer, 2018). Deficiency of maternal nutrients would exert a negative impact on fetal and postnatal performance due to intrauterine growth retardation and inefficiency in milk secretion (Kim et al., 2009; Kim and Wu, 2009; Zhang et al., 2019). Therefore, effective nutritional strategy is critical to improve sow and litter performance.

There is mounting evidence that maternal nutrition can elicit epigenetic modification of the fetal genome and expression of imprinted genes (Wu et al., 2004). Characteristics and variable patterns of epigenetic modification could cause changes in intestinal and muscle tissues under various environmental conditions (Jorgensen and Ro, 2019). The epigenetic modification represents a change not in the underlying deoxyribonucleic acid (**DNA**) sequence but in the way that how the cells read the genes. For epigenetic modification, DNA methylation and histone acetylation are caused by which methyl groups are transferred to DNA molecules and acetyl residue transferred to histone proteins, respectively, leading to a change of global gene expression (Sterner and Berger, 2000; Moore et al., 2013). According to Anderson et al. (2012), epigenetic gene regulation could be modified by nutritional influence, leading to changes in gene expression. Therefore, the enzyme cofactors and methyl donor might be expected to lead DNA methylation and histone acetylation with the changes in global gene expression and biological reactions (Waterland, 2006; Delage and Dashwood, 2008). A previous study showed that the supplementation of methionine analog as a methyl donor in diets improved the protein accretion and growth rate as well as an immune response in broiler chickens (Zhang and Guo, 2008). Therefore, DNA methylation and histone modifications can be altered by the overall availability of amino acids and micronutrients, which play an important role in regulating the availability of enzyme groups causing the epigenetic modification.

Zinc (Zn), Manganese (Mn), and Copper (Cu) are essential minerals for the embryonic and fetal development (Hostetler et al., 2003). Previous studies also showed that these trace minerals had key functional involvements in the intestinal immune system (Shannon and Hill, 2019) and muscle growth (Zhao et al., 2014) of pigs. Zinc enhanced intestinal barrier function with improved gene expression of claudin-1 and occludin in broiler chicken (Zhang et al., 2012). Supplementation of Mn could protect the chick embryo from stress conditions by enhancing antioxidant and antiapoptotic activation (Zhu et al., 2017). Supplementation of Cu has been related to influence embryo development, oxidase activity, and organ development in fetus (Fell et al., 1965; Grace et al., 1986; Gooneratne and Christensen, 1989).

Organic minerals are broadly used in the swine industry to enhance the bioavailability of minerals, reduce risks of heavy metal contamination, and reduce excretion to the environment (Hollis et al., 2005; Mateo et al., 2007; Mahan et al., 2014). Organic minerals include chelated or complexed forms with amino acids, organic acids, peptides, polysaccharides, and proteins (AAFCO, 2018). Liu et al. (2014) reported that mineral methionine hydroxy analog chelate (**MMHAC**) had a greater digestibility and retention of Zn, Cu, and Mn in growing pigs compared with trace mineral sulfates. According to Zhao et al. (2014), growing pigs fed with 80 mg/kg Cu-methionine hydroxy analog chelate (Cu-**MHAC**) in the diets had greater loin depth compared with 160 mg/kg CuSO_4_, indicating that less MMHAC could be used to replace high inorganic mineral in the diets. Supplementation of Zn-MHAC in broiler breeder diets reduced intestinal inflammation and improved intestinal integrity and immunity in progeny chicks by the upregulation of an anti-inflammatory gene, A20 protein, via reducing DNA methylation and increasing histone acetylation (Li et al., 2015). Considering the biological functions of Zn, Mn, and Cu in fetal and postnatal development as well as the biological efficacy of organic minerals, MMHAC could effectively enhance the development of the skeletal muscle and intestinal immunity of progeny by epigenetic modifications when fed to sows during pregnancy.

It is hypothesized that the supplementation of MMHAC in sow diets may enhance epigenetic modifications in skeletal and intestinal tissues in fetuses by enhancing muscle development and modulating intestinal inflammatory status. To test the hypothesis, the objective of this study was to determine the effects of MMHAC partially replacing Zn, Mn, and Cu sulfates in sow diets on sow and litter performance, global DNA methylation, histone acetylation, and gene expression related to muscle development and intestinal inflammation of progeny.

## Materials and Methods

The protocol for this study was reviewed and approved by the North Carolina State University Institutional Animal Care and Use Committee (Raleigh, NC). This study was conducted at a commercial swine farm (NG Purvis Farm, Carthage, NC).

### Animals, experimental design, and diets

A group of sows were bred and checked for the pregnancy at day 35 of gestation. Sixty pregnant sows (average parity: 3.8 ± 2.0) were assigned to two dietary treatments at day 35 in a randomized completed block design with parity of sows as a block (first and second parities vs. multiparities). The experimental diets included 0.02% mineral premix on each treatment. Treatments were 1) **ITM**: ZnSO_4_, MnO, and CuSO_4_ and (2) **CTM**: same Zn, Mn, and Cu concentrations as ITM treatment, but 50% of ITM was replaced with Zn-, Mn-, and Cu-MHAC (MINTREX Zn, Mn, and Cu, Novus International Inc., St Charles, MO; Table 1). The experimental diets contained corn and poultry fat as major energy feeds and soybean meal as a major protein supplement with supplemental amino acids, phytase, yeast, and choline. The dietary formulation is not shown to protect proprietary information of the N.G. Purvis Farm but the analyzed mineral composition in the diets is shown in Table 2. The inclusion levels of trace minerals met the NRC (2012) requirements.

**Table 1.** Micromineral concentration in dietary treatments^1^

	Treatments	
Mineral source, mg/kg	ITM	CTM
ZnSO_4_	125.0	62.5
Zn-MHAC	0.0	62.5
MnO	40.0	20.0
Mn-MHAC	0.0	20.0
CuSO_4_	16.0	8.0
Cu-MHAC	0.0	8.0

^1^The same amount of methionine provided by organic minerals as a form of Zn-MHAC, Mn-MHAC, and Cu-MHAC (MINTREX, Novus International, St. Charles, MO) was also supplemented as a form of MHA in the ITM diet.

**Table 2.** Analyzed mineral concentrations in diets (as-fed basis)

Item	ITM^1^	CTM^2^
Analyzed composition		
Gestation diet		
Zn, mg/kg	192.7	173.7
Mn, mg/kg	89.3	81.3
Cu, mg/kg	29.3	20.7
Ca, %	0.83	0.84
Total P, %	0.78	0.78
Lactation diet		
Zn, mg/kg	208.5	182.5
Mn, mg/kg	91.0	92.0
Cu, mg/kg	52.0	34.5
Ca, %	1.09	1.07
Total P, %	0.66	0.65

^1^ITM, conventional inorganic sources of trace minerals (0.2% inclusion level in the diets).

^2^CTM, 50:50 MMHAC and inorganic minerals (0.2% inclusion level in the diets).

According to standard operating procedures of the commercial farm, all sows received assigned gestation diets at 2 kg/d until farrowing regardless of their body weight (**BW**) or parity. Pregnant sows were weighed and moved to a farrowing building on day 109 of gestation. Upon farrowing, sows were given ad libitum access to experimental diets until weaning (average day 21 of lactation). Feed intake of sows was recorded daily. All piglets were weighed at birth, on day 9, and on day 18 of lactation. Sows were weighed on day 18 of lactation.

### Sample collection and preparation

Upon farrowing, the 1 male piglet representing the fourth or fifth birth of 32 litters (16 piglets per treatment) was separated for blood and tissue sampling before approaching the colostrum to determine the effects of MMHAC supplementation during gestation. For this study, only the male piglets were selected to determine the effects of MMHAC supplementation on the measures behind unexpectable sex effects (Tarleton et al., 2001; Jiao et al., 2009; Haren et al., 2011). Blood sampling was conducted with BD sterile vacutainers (BD, Franklin Lakes, NJ) to collect serum, and then the piglets were euthanized to collect jejunal and muscle tissues. If both fourth and fifth piglets were female, the next male pig was selected for sampling. Colostrum and milk samples were collected from the third, fourth, and fifth pairs of mammary glands after intravulval injection of 1 mL oxytocin (5 to 20 IU) within 24 h of farrowing and on day 18 of lactation. Upon weaning on day 18, male piglets representing a median BW of each litter were selected for blood sampling, and then the piglets were euthanized to collect jejunal and muscle tissues. Blood samples were centrifuged to separate serum and stored at −80 °C until analysis. Upon euthanasia, jejunal tissues were separated from mid-jejunum at 1 m (at farrowing) or 3 m (on day 18 of lactation) after the duodenojejunal junction. Lumen content was removed by gentle washing with sterilized saline solution. A part (3 cm) was placed in a 50-mL tube with 10% buffered formaldehyde for fixation and immunohistochemistry. Another two parts (3 cm each) were stored in liquid nitrogen and stored at −80 °C for DNA methylation and histone acetylation measurements. Another part (1 cm) was collected and stored in 1.2 mL RNALater (Thermo Fisher Scientific Inc., Rockford, IL, USA) using 2 mL tube for 24 h at 4 °C and then stored at −20 °C until analysis for gene expression. The remaining part (9 cm) was used to collect mucosal tissues and then stored at −80 °C for cytokines measurement. Two samples of muscle were collected from the left *longissimus dorsi* of carcass between 10th and 11th ribs. *Longissimus dorsi* muscle is the part having accuracy and precision to assess the body and carcass composition of the carcass (Gresham et al., 1994). One was frozen in liquid N and stored at −80 °C until analysis for DNA methylation and histone acetylation measurements. The other sample (~0.5 cm in all three dimensions) was collected and stored in 1.2 mL RNALater using 2 mL tube for 24 h at 4 °C and then stored at −20 °C until analysis for gene expression.

### Mineral composition in colostrum and milk

Colostrum and milk samples were stored at −20 °C and freeze-dried (24D 48, Virtis, Gardiner, NY, USA). The dried milk samples were submitted to the Environmental and Agricultural Testing Service (EATS) laboratory, Department of Crop and Soil Sciences, at North Carolina State University. The minerals (Zn, Mn, Cu, and Ca) concertation in colostrum and milk samples was determined using atomic absorption spectrometry (Perkin-Elmer 3100, Shelton, CT)

### Morphological evaluation and immunohistochemistry

Jejunal tissue samples were fixed in 10% formalin buffer for 3 wk and sent to the Histology Laboratory of North Carolina State University (Raleigh, NC, USA) for hematoxylin and eosin staining as well as immunohistochemistry for detecting Ki67^+^ antibody as a biological marker for measuring the proliferation of enterocytes. A total of 15 villus and 15 crypts in each slide were selected to measure villus height (**VH**), villus width, crypt depth (**CD**), and percent of Ki67^+^ enterocyte using a microscope (Olympus CX31 microscope, Tokyo, Japan). The ratio of VH to CD (**VH:CD**) was calculated. The histomorphology was measured as previously described by Duarte et al. (2019).

Immunoglobulins concentration was analyzed using the method described by Shen et al. (2011). Total concentrations of immunoglobulin G (**IgG**), immunoglobulin A (**IgA**), and immunoglobulin M (**IgM**) in the colostrum and milk of sows, as well as in the jejunal mucosa and serum of nursery pigs, were measured by enzyme-linked immunosorbent assay (**ELISA**) according to the manufacturer’s protocols (Bethyl Laboratories Inc., Montgomery, TX). Before analysis, all samples of colostrum, milk, and mucosal tissue were diluted to 1:800,000, 1:50,000, and 1:1,600 to measure IgG, IgA, and IgM, respectively.

### IgG, IgA, and IgM

Immunoglobulins concentration was analyzed using the method described by Shen et al. (2011). Total concentrations of IgG, IgA, and IgM in the colostrum and milk of sows, as well as in the jejunal mucosa and serum of nursery pigs, were measured by ELISA according to the manufacturer’s protocols (Bethyl Laboratories Inc., Montgomery, TX). Jejunal mucosa samples were weighed and suspended into 1.0 mL phosphate-buffered saline. The suspended samples were homogenized on ice (Tissuemiser, Thermo Fisher Scientific Inc., Rockford, IL, USA). The homogenate was centrifuged at 14,000 × *g* for 20 min. The supernatant was used to determine concentrations of total protein in jejunal mucosa and muscle for analysis. Total protein concentration in the mucosa was determined by using the Pierce BCA Protein Assay Kit (Thermo Fisher Scientific Inc. Rockford, IL, USA). The colostrum, milk, and mucosal tissue homogenate were diluted to 1:800,000, 1:50,000, and 1:1,600 to measure IgG, IgA, and IgM, respectively.

### ELISA for tumor necrosis factor-alpha, interleukin-8, transforming growth factor-beta 1, mucin-2, and myogenic factor 6

Sample preparation for analysis was followed as previously described by Chen et al. (2017). Jejunal mucosa and muscle samples were weighed and suspended into 1.0 mL phosphate-buffered saline. The suspended samples were homogenized on ice (Tissuemiser, Thermo Fisher Scientific Inc., Rockford, IL, USA). The homogenate was centrifuged at 14,000 × *g* for 20 min. The supernatant was used to determine the concentrations of total protein in the jejunal mucosa and muscle for analysis. Total protein concentration in mucosa and muscle samples was determined by using the Pierce BCA Protein Assay Kit (Thermo Fisher Scientific Inc., Rockford, IL, USA). The concentration of tumor necrosis factor-alpha (**TNF-α**) in serum and jejunal mucosa was measured by ELISA (R&D Systems, Minneapolis, MN) according to the manufacturer’s protocol. The concentration of interleukin-8 (**IL-8**), transforming growth factor-beta-1 (**TGF-β1**), mucin-2 (**MUC2**) protein in jejunal mucosa, and myogenic factor 6 (**MYF6**) in *longissimus* muscle were measured by ELISA (MyBioSource, San Diego, CA) according to the manufacturer’s protocols.

### Global DNA methylation and histone acetylation

Genomic DNA was isolated from jejunum or *longissimus* muscle using Trizol reagent (Thermo Fisher Scientific Inc., Rockford, IL, USA). A total of 3 ng of genomic DNA from jejunum or *longissimus* muscle of each pig were used for quantitation of methylation using The MethylFlashTM Methylated DNA Quantification Kit (Epigentek, Farmingdale, NY). Methylation (%) was calculated as methylated DNA (ng)/input DNA (3 ng) × 100. Total histone was extracted from jejunum or *longissimus* muscle using the EpiXtract Total Histone Extraction Kit (Epigentek, Farmingdale, NY). Protein concentration of total histone was measured using the Pierce BCA Protein Assay Kit (Thermo Fisher Scientific Inc., Rockford, IL, USA); 50 ng of histone protein from *longissimus* muscle or 180 ng of histone protein from jejunum was used to measure histone acetylation using EpiQuik Global Acetyl Histone H3-K9 Quantification Kit (Epigentek, Farmingdale, NY). Acetylated histone was expressed as µg/mg protein as described in the manufacturer’s protocol.

### Quantification of gene expression

Total ribonucleic acid (**RNA**) was isolated from the jejunum or *longissimus* muscle using Trizol reagent (Thermo Fisher Scientific Inc., Rockford, IL, USA); 1 µg of total RNA was used to synthesize complementary DNA using oligo dT and M-MLV Reverse Transcriptase (Thermo Fisher Scientific Inc., Rockford, IL, USA) according to the manufacturers’ instructions. Relative levels of messenger ribonucleic acid (**mRNA**) were measured by quantitative real-time polymerase chain reaction (**PCR**) using Applied Biosystems SYBR Green PCR Master Mix (Thermo Fisher Scientific Inc., Rockford, IL, USA) and a QS5 Real-Time PCR System. Results were expressed as the level relative to the corresponding housekeeping gene *beta*-*actin*. The Ct of housekeeping gene *beta*-*actin* was not statistically affected by dietary treatment. Therefore, *beta-actin* was chosen as a housekeeping gene for this study and quantified along with each gene, and the relative expression of each gene was normalized to *beta-actin* using delta–delta–Ct method as described previously (Livak and Schmittgen, 2001) *and* expressed as the level relative to *beta-actin*. All primers (Table 3) were verified for melting curve, efficiency (100% ± 10%), and linearity (*r*^2^ ≥ 0.99) of amplification.

**Table 3.** Sequence of primers for jejunum and skeletal muscle

Item	Forward primers	Reverse primers	Accession number
MEF2C	GACATCGTGGAGACGTTGAGA	TCAGGGCTGTGACCTACTGAA	NM_001044540.1
MSTN	CATGAACCCAGGCACTGGTA	TCCTGGTCCTGGGAAGGTTA	NM_214435.2
MYOD1	GACGGCACCTATTACAGCGA	CACGATGCTGGACAGACAGT	NM_001002824.1
MYF5	AGTTCGGGGACGAGTTTGAG	GTGGATTTCCTCTTGCACGC	KC456667.1
MRF4	CTCAGGAGCTCACGAAAGGG	CCACCCAGCAAAAACCAAGG	NM_001244672.1
MEF2A	CATGGCAAACAGAGCACCCT	TGCAAGCTAGTGTGGGAGAA	NM_001097421.1
DEGS1	ATGGCATCGACGTGGATATTC	GCATAAAAGAGCGGCTGAAGA	NM_001244121.**1**
MTOR	CAAACCCCGGAGTGATCAAC	ATAATAAAAAGTTCATCCACCCACTTC	XM_003127584.6
NF-κB (P50)	TTGAAACACTGGAAGCACGAA	CCACCTTCCGCTTGCAAATA	KC316024.1
NF-κB (P105)	TGGCAGTGATCACCAAGCA	CAGCGAGGTGCAAAACAGAGT	KC316025.1
IL-8	AAGCTTGTCAATGGAAAAGAG	CTGTTGTTGTTGCTTCTCAG	AB057440.1
MUC2	CAACGGCCTCTCCTTCTCTGT	GCCACACTGGCCCTTTGT	XM_021082584.1
TGFβ-1	TGACCCGCAGAGAGGCTATAG	GGCCAGAATTGAACCCGTTA	XM_021093503.**1**
TNF-α	GCCGTCTCCTACCAGACCAA	CTCTGGCAAGGGCTCTTGAT	JF831365.1
Beta-actin	CAAATGCTTCTAGGCGGACTGT	TCTCATTTTCTGCGCAACTTAGG	XM_003124280.5

### Statistical analysis

Data from this study were analyzed based on a randomized complete block design by the mixed model of SAS Software (Cary, NC). Sow was considered as an experimental unit for reproductive performance parameters, and piglet was the experimental unit for other parameters. Treatment was the fixed effects and the parity block was a random effect. The statistical difference among treatment means was considered significant with *P* < 0.05, whereas 0.05 ≤ *P* < 0.10 was considered as tendency.

## Results

### Sow and litter performance

Average parity of sow was not different between treatments (Table 4). The BW of sows at days 50 and 110 of gestation and BW change of sow during gestation were not different between treatments (Table 5). BW of sows, average daily feed intake (**ADFI**), litter size, and litter weight during lactation were not different between treatments (Table 6). Sows fed a diet with MMHAC tended to have decreased (*P* = 0.059) BW loss during lactation compared with sows fed a diet with ITM. The BW of piglets at birth was not different between treatments. Piglets from sows fed a diet with MMHAC tended to have increased (*P* = 0.098) BW of piglets on day 18 after birth. The concentration of Zn, Mn, Cu, and Ca in colostrum and milk were not affected by the supplementation of MMHAC in sow diets when measured on days 1 and 18 of lactation (Table 7).

**Table 4.** Reproductive performance of sows in the previous parity

Item	ITM^1^	CTM^2^	SEM	*P*-value
*N*	24	23		
Gestation period	115.5	115.7	0.2	0.447
Sow parity	3.33	3.61	0.35	0.577
Litter size, head				
At birth, total	14.2	14.7	0.9	0.689
At birth, live	13.4	13.4	0.8	0.996
Stillborn	0.5	0.7	0.2	0.579
Mummies	0.3	0.6	0.2	0.292

^1^ITM, conventional inorganic sources of trace minerals (0.2% inclusion level in the diets).

^2^CTM, 50:50 MMHAC and inorganic minerals (0.2% inclusion level in the diets).

**Table 5.** Supplemental effects of MMHAC in sow diets on the performance of sows during gestation

Item	ITM^1^	CTM^2^	SEM	*P*-value
*N*	29	28		
Sow parity	3.79	3.96	0.38	0.703
BW of sows, kg				
Day 50 of gestation	238.4	240.8	5.5	0.738
Day 110 of gestation	278.2	274.8	6.1	0.663
BW change, kg				
Day 50 to 110 of gestation	39.9	34.0	3.2	0.184

^1^ITM, conventional inorganic sources of trace minerals (0.2% inclusion level in the diets).

^2^CTM, 50:50 MMHAC and inorganic minerals (0.2% inclusion level in the diets).

**Table 6.** Supplemental effects of MMHAC in sow diets on the performance of sows and litters during lactation

Item	ITM^1^	CTM^2^	SEM	*P-*value
*N*	28	25		
Sow parity	3.82	3.84	0.40	0.973
BW of sows, kg				
Day 50 of gestation	238.4	240.8	5.5	0.738
Day 110 of gestation	278.2	274.8	6.1	0.663
Day 1 of lactation^3^	261.0	257.8	5.3	0.635
Day 20 of lactation (at weaning)	244.1	250.9	5.1	0.314
BW change, kg				
Day 50 to 110 of gestation	39.9	34.0	3.2	0.184
Day 1 to weaning	−16.9	−6.9	3.5	0.059
ADFI, kg	6.19	6.45	0.19	0.282
Litter size, head				
At birth, total	14.4	14.9	0.7	0.583
At birth, live	13.8	13.6	0.7	0.832
Stillborn	0.5	0.8	0.3	0.543
Mummies	0.1	0.5	0.2	0.128
Day 9	11.1	10.7	0.4	0.521
Day 18	10.9	10.2	0.5	0.260
Litter weight, kg				
At birth, total	19.2	18.3	1.1	0.612
At birth, live	18.4	17.3	1.1	0.482
Day 9	33.2	33.6	1.6	0.843
Day 18	58.0	58.1	2.5	0.991
Litter BW gain	39.6	40.7	2.1	0.719
Piglet BW, kg				
At Birth	1.35	1.28	0.04	0.335
Day 9	3.00	3.15	0.09	0.256
Day 18	5.34	5.75	0.15	0.098
Piglet mortality, %	19.3	24.1	2.6	0.231

^1^ITM, conventional inorganic sources of trace minerals (0.2% inclusion level in the diets).

^2^CTM, 50:50 MMHAC and inorganic minerals (0.2% inclusion level in the diets).

^3^BW on lactation day 1 was calculated as BW on gestation day 110 subtracted from the litter weight.

**Table 7.** Supplemental effects of MMHAC in sow diets on mineral composition in colostrum and milk (DM basis)

Item	ITM^1^	CTM^2^	SEM	*P-*value
*N*	16	16		
Sow parity	3.62	3.62	0.46	1.000
Colostrum				
Zn, mg/kg	69.53	70.21	2.86	0.863
Mn, mg/kg	0.23	0.14	0.06	0.327
Cu, mg/kg	18.24	15.68	1.07	0.121
Ca, %	0.22	0.20	0.01	0.191
Milk, day 18 of lactation				
Zn, mg/kg	31.05	29.28	1.52	0.411
Mn, mg/kg	0.55	0.52	0.07	0.801
Cu, mg/kg	6.36	6.58	0.27	0.567
Ca, %	1.04	0.93	0.04	0.104

^1^ITM, conventional inorganic sources of trace minerals (0.2% inclusion level in the diets).

^2^CTM, 50:50 MMHAC and inorganic minerals (0.2% inclusion level in the diets).

### Intestinal health, global DNA methylation, histone acetylation, and gene expression in the jejunum and *longissimus* muscle of piglets

No differences in jejunal histomorphology and crypt cell proliferation of piglets were observed between treatments on days 1 and 18 of lactation (Table 8). Concentration of IgG, IgA, and IgM in colostrum, milk, serum, and jejunal mucosa of piglets did not differ between treatments on days 1 and 18 of lactation. (Table 9). Concentration of TNF-α, IL-8, TGF-β1, and MUC2 in the jejunal mucosa and myogenic regulatory factor 4 (**MRF4**) in the muscle of the piglets were not affected by the supplementation of MMHAC in sow diets on days 1 and 18 of lactation (Table 10).

**Table 8.** Supplemental effects of MMHAC in sow diets on morphology and crypt cell proliferation in the jejunum of suckling piglets

Item	ITM^1^	CTM^2^	SEM	*P*-value
Jejunum, day 1 of lactation				
VH, μm	690	651	36	0.446
Villus width, μm	70	71	2	0.891
CD, μm	94	94	5	0.996
VH:CD	8.92	8.67	0.33	0.600
Crypt cell proliferation, %	42.3	46.1	2.9	0.369
Jejunum, day 18 of lactation				
VH, μm	488	422	51	0.394
Villus width, μm	88	89	2	0.908
CD, μm	162	151	7	0.282
VH:CD	3.24	3.44	0.21	0.493
Crypt cell proliferation, %	37.3	36.2	4.6	0.874

^1^ITM, conventional inorganic sources of trace minerals (0.2% inclusion level in the diets).

^2^CTM, 50:50 MMHAC and inorganic minerals (0.2% inclusion level in the diets).

**Table 9.** Supplemental effects of MMHAC in sow diets on immunoglobulin concentration in colostrum and milk of sows and serum and jejunal mucosa of suckling piglets

Item	ITM^1^	CTM^2^	SEM	*P-*value
Colostrum, day 1 of lactation				
IgG, mg/mL	118.68	111.95	22.42	0.832
IgA, mg/mL	18.20	17.34	2.72	0.819
IgM, mg/mL	8.68	7.79	1.41	0.648
Milk, day 18 of lactation				
IgG, mg/mL	2.32	2.02	0.34	0.503
IgA, mg/mL	4.69	5.14	0.32	0.331
IgM, mg/mL	2.92	2.81	0.29	0.779
Serum, day 1 of lactation				
IgG, mg/mL	0.139	0.129	0.025	0.762
IgA, mg/mL	0.008	0.006	0.001	0.262
IgM, mg/mL	0.064	0.060	0.005	0.614
Serum, day 18 of lactation				
IgG, mg/mL	2.966	2.717	0.192	0.339
IgA, mg/mL	0.175	0.132	0.020	0.164
IgM, mg/mL	0.938	1.037	0.129	0.580
Jejunal mucosa, day 1 of lactation				
IgG, mg/g	2.501	1.942	0.307	0.226
IgA, mg/g	0.605	0.558	0.032	0.307
IgM, mg/g	0.220	0.190	0.032	0.518
Jejunal mucosa, day 18 of lactation				
IgG, mg/g	7.780	8.643	1.109	0.580
IgA, mg/g	5.025	5.185	1.467	0.939
IgM, mg/g	4.461	4.572	0.782	0.920

^1^ITM, conventional inorganic sources of trace minerals (0.2% inclusion level in the diets).

^2^CTM, 50:50 MMHAC and inorganic minerals (0.2% inclusion level in the diets).

**Table 10.** Supplemental effects of MMHAC in sow diets on inflammatory cytokines, MUC, and MYF in the jejunum and *longissimus* muscle of suckling piglets

Item	ITM^1^	CTM^2^	SEM	*P-*value
TNF-α in jejunal mucosa, pg/mg				
Day 1 of lactation	0.97	0.80	0.08	0.168
Day 18 of lactation	1.21	1.24	0.12	0.896
IL-8 in jejunal mucosa, pg/mg				
Day 1 of lactation	3.75	2.89	0.82	0.451
Day 18 of lactation	34.90	27.51	3.99	0.211
TGF-β1 in jejunal mucosa, pg/mg				
Day 1 of lactation	35.08	26.26	6.65	0.360
Day 18 of lactation	7.65	7.49	0.79	0.884
MUC2 in jejunal mucosa, U/mg				
Day 1 of lactation	0.86	0.85	0.23	0.996
Day 18 of lactation	0.40	0.43	0.04	0.601
MYF6 in muscle, ng/mg				
Day 1 of lactation	0.45	0.41	0.06	0.665
Day 18 of lactation	0.21	0.23	0.02	0.464

^1^ITM, conventional inorganic sources of trace minerals (0.2% inclusion level in the diets).

^2^CTM, 50:50 MMHAC and inorganic minerals (0.2% inclusion level in the diets).

Piglets from sows fed a diet with MMHAC had a higher (*P* < 0.05) status of global acetylation in the muscle tissue at birth than piglets from sows fed diet without MMHAC (Table 11). Expression of nuclear factor kappa B (**NF-κB**) P50 subunit and *NF-κB* P105 mRNA as a precursor of NF-κB, one of the key regulators of intestinal inflammation (Neurath et al., 1998; Shifera, 2010), were downregulated (*P* < 0.05) in the jejunal mucosa of piglets from sow fed with MMHAC on day 18 of lactation (Table 12). Expression of *MUC2* (*P* = 0.077) and *TGF-β1* (*P* = 0.057) tended to be downregulated in the jejunum of piglets by the supplementation of MMHAC on day 18 of lactation. Muscle development-related genes were measured in *longissimus* muscle (Table 13). Expression of *MRF4* mRNA tended to be decreased (*P* = 0.068) at birth in the muscle of piglets from sows fed with MMHAC during gestation. Expression of delta 4-desaturase sphingolipid1 (**DEGS1**) mRNA tended to be increased (*P* < 0.10) at birth and was decreased (*P* < 0.05) on day 18 of lactation in the muscle of piglets from sow fed with MMHAC.

**Table 11.** Supplemental effects of MMHAC in sow diets on global methylation and acetylation in the jejunum and *longissimus* muscle of suckling piglets

Item	ITM^1^	CTM^2^	SEM	*P-*value
Day 1 of lactation				
Jejunum				
Methylation	7.8	6.7	0.8	0.303
Acetylation	360.8	343.6	13.8	0.383
Muscle				
Methylation	11.6	11.6	1.8	0.999
Acetylation	748.9	1,137.4	112.7	0.021
Day 18 of lactation				
Jejunum				
Methylation	13.4	14.9	1.2	0.378
Acetylation	338.6	305.8	32.7	0.484
Muscle				
Methylation	9.7	8.6	2.1	0.705
Acetylation	75.8	75.1	1.0	0.646

^1^ITM, conventional inorganic sources of trace minerals (0.2% inclusion level in the diets).

^2^CTM, 50:50 MMHAC and inorganic minerals (0.2% inclusion level in the diets).

**Table 12.** Supplemental effects of MMHAC in sow diets on the expression of key mRNA related to jejunal inflammation in suckling piglets

Item	ITM^1^	CTM^2^	SEM	*P-*value
Day 1 of lactation, × 10^–5^				
*IL-8*	1,344	1,018	178	0.193
*MUC2*	5,380	5,511	984	0.925
*NF-κB (p50)*	701	693	93	0.944
*NF-κB (p105)*	1,991	1,646	211	0.274
*TGF-β1*	2,000	1,600	370	0.500
*TNF-α*	11	7	2	0.174
Day 18 of lactation, × 10^–5^				
*IL-8*	1,134	787	220	0.262
*MUC2*	5,773	3,871	722	0.077
*NF-κB (p50)*	752	507	84	0.048
*NF-κB (p105)*	1,589	995	172	0.012
*TGF-β1*	2,000	1,400	230	0.057
*TNF-α*	21	27	3	0.139

^1^ITM, conventional inorganic sources of trace minerals (0.2% inclusion level in the diets).

^2^CTM, 50:50 MMHAC and inorganic minerals (0.2% inclusion level in the diets).

**Table 13.** Supplemental effects of MMHAC in sow diets on the expression of key mRNA related to muscle development of suckling piglets

Item	ITM^1^	CTM^2^	SEM	*P-*value
Day 1 of lactation, × 10^–6^				
MEF2C	312	299	48	0.838
MSTN	58	54	10	0.770
MYOD1	218	165	43	0.375
MYF5	101	134	18	0.191
MRF4	3,529	2,436	408	0.068
MEF2A	1,166	910	128	0.157
DEGS1	19,870	26,424	2,599	0.086
MTOR	88	80	21	0.798
Day 18 of lactation, × 10^–6^				
MEF2C	825	618	126	0.249
MSTN	558	504	71	0.591
MYOD1	1,385	995	169	0.114
MYF5	241	225	24	0.636
MRF4	9,207	10,555	1,091	0.390
MEF2A	3,047	2,506	280	0.175
DEGS1	54,844	33,777	4,182	0.001
MTOR	195	298	47	0.130

^1^ITM, conventional inorganic sources of trace minerals (0.2% inclusion level in the diets).

^2^CTM, 50:50 MMHAC and inorganic minerals (0.2% inclusion level in the diets).

## Discussion

This study demonstrated that dietary supplementation of MMHAC replacing inorganic minerals benefited sows by reducing BW losses during lactation and suckling piglets by increasing BW at weaning. The NF-κB is a signaling molecule that plays a key role in the regulation of inflammation pathway; it can be activated by external stimuli such as pathogens and inflammation and then translocate into the nucleus and stimulate the expression of proinflammatory cytokines, such as *IL-1β, IL-6, IL-8, IL-12, IL-18, TNF-α,* and interferon-gamma. In this study, the expression of *NF-κB* P50 subunit and P105 precursor were significantly reduced in piglets from sows fed MMHAC; however, the expression of *TNF-α* and *IL-8* were not reduced, NF-κB may downregulate other proinflammatory cytokines that were not measured in this study, indicating that maternal supplementation of MMHAC probably reduced intestinal inflammation in progeny piglet. Regulation of muscle-related gene expression indicated that MMHAC increased muscle fiber hypertrophy in suckling piglets. These outcomes partially explain how MMHAC reduced BW loss of sows and increased BW of suckling piglets. Results from this study indicated that genes related to muscle development at birth and intestinal health at weaning were modulated by feeding a diet with MMHAC.

The differentiation of muscle is regulated by a group of *MRF*, including myogenic differentiation 1 (**MYOD**1), myogenic factor 5 (**MYF**5), myogenin (**MYOG**), and MRF4 (Moncaut et al., 2013; Moretti et al., 2016). The *MYOD1* and *MYF5* are involved in the generation of myogenic precursors called myoblasts; *MYOG* and *MRF4* are required for terminal differentiation of committed muscle progenitors into myofibers (Moncaut et al., 2013). The expression of *MRF4* starts during fetal development, continues throughout postnatal stages, and is the predominant *MRF* in adult skeletal muscle (Hinterberger et al., 1991; Yan et al., 2013), suggesting that *MRF4* may regulate skeletal muscle maturation and hypertrophy during late fetal development and postnatal muscle growth. Previous studies showed that the knockdown of *MRF4* expression at both gene and protein levels induced muscle fiber hypertrophy, increased protein synthesis, and activated expression of a variety of muscle-specific genes in skeletal muscle, indicating that *MRF4* negatively regulates skeletal muscle growth (Moretti et al., 2016; Schiaffino et al., 2018). In addition, the overexpression of *MRF4* in a line of transgenic mice reduced the skeletal muscle fiber growth during regeneration (Pavlath et al., 2003). These findings suggest that *MRF4* acts as a negative regulator of muscle hypertrophy. Therefore, lower expression of *MRF4* could enhance muscle hypertrophy in skeletal muscle. In this study, maternal supplementation of MMHAC tended to reduce (*P* = 0.068) *MRF4* expression at birth in the muscle of piglets from sow fed with MMHAC during gestation, suggesting that MMHAC probably plays some roles in promoting muscle hypertrophy in skeletal muscle. This might be part of the mechanisms by which supplementation of MMHAC increased loin eye area in their pigs in comparison to progeny pigs from ITM sows and supplementation of 80 and 160 mg/kg chelated Cu as Cu-MHAC increased loin depth in grower-finisher pigs compared with 160 mg/kg CuSO_4_ (Zhao et al., 2014). 

In this study, MMHAC increased global histone acetylation in the *longissimus* muscle of piglets at birth, indicating that MMHAC potentially increased the expression of genes that are related to muscle development in the fetus during gestation. During gestation, the number of muscle fibers in pigs is predetermined through embryonic development (Wigmore and Stickland, 1983). After myogenesis, muscle growth is achieved by muscle fiber hypertrophy (Dwyer et al., 1994; Murray et al., 2018). Yan et al. (2013) reported the impact of maternal nutrition on the fetal development in pigs, especially related to fetal skeletal muscle and intestine development. In particular, maternal nutrition during the embryonic stage has relatively minor effects on skeletal muscle development, because only a very small number of myofibers would be formed during early gestation. Dwyer et al. (1994) indicated that the critical stage for fetal skeletal muscle development is mid-to-late gestation in pigs.

Histone H3K9 acetylation is a primary acetylated site of histone H3 and an active chromatin epigenetic tag (Turner, 2000). Histone acetylation modulated by histone acetyltransferases and histone deacetylases (**HDAC**) can be one marker of epigenetic modifications, which can be associated with the regulation of the muscle-specific genes transcription, repression, and activation (Moresi et al., 2015). The HDAC inhibitors, which inhibit histone deacetylation, could ultimately increase histone acetylation, thereby regulating gene expression. These HDAC inhibitors increased *MyoD* acetylation in myoblasts (Lezzi et al., 2002), myocyte enhancer factor 2 (**MEF**2) acetylation in human embryonic kidney 293 (HEK293) cells (Grégoire et al., 2007), and histone H3 acetylation, which leads to the upregulation of cardiac gene expression in cardiomyocytes (Otsuji et al., 2012). The HDAC inhibitors have also been reported to increase myofiber size and alleviate muscle loss and dysfunction in mice (Minetti et al., 2006) and to enhance muscle regeneration for treatment of muscular dystrophy (Sincennes et al., 2016). Dietary supplementation of tributyrin, one of HDAC inhibitors, during neonatal phase increased *longissimus* muscle DNA to protein ratio, muscle fiber cross-sectional area, and loin eye area in the piglets, suggesting that an increase of histone acetylation with HDAC inhibitor promotes *longissimus* muscle growth (Murray et al., 2018). These findings support that the increase of histone acetylation could enhance skeletal muscle growth and regeneration by improving hypertrophy. In this study, only global histone H3K9 acetylation and DNA methylation were measured. In addition, epigenetic changes were not measured in the loci where gene expression was measured. Due to these limitations, the results from this study do not fully support whether any of the changes in gene expression are due to epigenetic modification. For the complete understanding, it warrants further investigation including chromatin immunoprecipitation assay. Based on findings in the literature, the increase of global histone acetylation in *longissimus* muscle suggests that supplementation of MMHAC in sows could potentially enhance skeletal muscle growth by upregulating the expression of muscle-associated genes in progeny piglets. However, which genes are modulated by histone acetylation and the detailed mechanism behind remains to be elucidated in future studies.

In addition, gene expression of *DEGS1* was increased at birth in the muscle of piglets from sows fed MMHAC during gestation. *DEGS1* gene is associated with the loin area and intramuscular fat deposition (Ropka-Molik et al., 2018). Decrease of *DEGS1* expression inhibited adipose cell proliferation, impaired lipid accumulation, and was correlated with fat mass (Barbarroja et al., 2015), which means that lower expression of *DEGS1* indicated lower levels of intramuscular fat. Piglets from sows fed with MMHAC had a tendency of higher expression of *DEGS1* at birth, indicating that piglets at birth could have higher intramuscular fat, which might help newly born piglets to maintain their energy. Moreover, gene expression of *DEGS1* was decreased in piglets from sows fed with MMHAC on day 18 of lactation. It has been reported that high feed efficiency of pigs had a lower proportion of intramuscular fat compared with the pigs having low feed efficiency (Horodyska et al., 2018). In this study, piglets at weaning from sows fed with MMHAC had lower expression of *DEGS1* mRNA, suggesting that they may have a lower percentage of intramuscular fat and higher feed efficiency. Therefore, this study indicates that maternal MMHAC may help the energy utilization of their piglets with differential regulation of *DEGS1* mRNA expression at birth and weaning.

It has been well-documented that trace minerals, especially, Zn, Mn, and Cu, are required for embryonic and fetal development because they have a significant role with high concentration accumulation in the embryo of uterus compared with other reproductive tissues (Hostetler et al., 2003). However, there is limited information for relationships between fetal muscle development, minerals bioavailability, and transfer mechanisms of these micronutrients. Among trace minerals, however, Zn, Mn, and Cu have potential common pathways for pregnancy by controlling the secretion of reproductive hormones, peroxidase activity, and embryonic development (Hostetler et al., 2003). In terms of gene expression, Zn can be a mediator on DNA and RNA transcription by the formation of Zn-finger proteins (**ZNF**), which are required for interaction with DNA, RNA, and other proteins, leading to the regulation of cellular processes (Hostetler et al., 2003; Cassandri et al., 2017). Previous studies showed that ZNF play a critical role in myogenic synthesis and differentiation with recruiting muscle-specific target genes (Cassandri et al., 2017; Nie et al., 2019). Salmon et al. (2009) showed that overexpression of ZNF could promote myogenic proliferation and inhibit differentiation. Zinc could help recruit muscle-specific target genes via ZNF, which will be the subject of future study. Maternal nutrition has been reported to play a significant role in fetal programming and epigenome (Gartstein and Skinner, 2018). For example, external or internal environmental factors such as pathogens and nutrition could alter epigenetic modifications and gene expression and define specific phenotypes (Ibeagha-Awemu and Zhao, 2015). Supplementation with folic acid during pregnancy or after weaning alters the phenotype and epigenotype of progeny induced by maternal dietary protein restriction during gestation (Burdge and Lillycrop, 2010; Perera and Herbstman, 2011). Based on the data from this study, it can be speculated that maternal supplementation of MMHAC helped the development of fetus with a potential increase of muscle fiber hypertrophy by the tendency of downregulation of *MRF4* gene expression and significant upregulation of global histone acetylation.

This study also shows that weaning piglets from sows fed a diet with MMHAC had lower expressions of *NF-κB, MUC2*, and *TGF-β1* in the jejunum compared with piglets from sows fed a diet with ITM. The *NF-κB* is a transcription factor responsible for immune and inflammatory response. The *MUC2* can be an indicator of the production of intestinal mucus (Johansson et al., 2011). Reduced *MUC2* expression was not correlated with impaired intestinal barrier functions when considering decreased *NF-κB* gene expression and increased growth of piglets at day 18 of lactation. Therefore, reduced expression of *MUC2* in piglets from sows fed a diet with MMHAC could also indicate that nutrients to make mucin could be saved potentially directing more nutrients for the growth of their piglets. Previous studies showed that chelated mineral sources can regulate intestinal inflammatory response (Liao et al., 2018; Shannon and Hill, 2019). The intestinal inflammatory response can be caused by various stimuli, such as viral antigen, bacteria invasion, cytokines, stress, and free radicals. Prasad et al. (2004) described that Zn had anti-inflammatory effect by decreasing gene expression of *TNF-α* and *IL-1β* through inhibiting *NF-κB* activation. Zinc amino acid complex supplementation reduced TNF-α levels in the pigs (Mayorga et al., 2018). Supplementation of Zn-MHAC in the breeder diets induced the reduction of intestinal inflammation in their progeny chicks compared with ZnSO_4_ supplementation (Li et al., 2015). Zhang et al. (2017) also showed that Cu regulated inflammatory cytokines in serum and altered intestinal microbiota in the rats. It has been demonstrated that copper proteinate reduced duodenal CD in early weaned pigs (Zhao et al., 2007) indicating a slower turnover of enterocytes and better intestinal integrity. These findings suggest that chelated or organic Zn and Cu play essential roles in the regulation of inflammatory response in the intestine. In this study, in which all three minerals, Zn, Mn, and Cu, that are chelated to methionine-hydroxy analog, were supplemented to the sows, which specific mineral(s) accounted for the reduction of intestinal inflammation is warranted to further investigate in future studies. It is possible that all three chelated minerals, Zn, Mn, and Cu, play important roles in the regulation of intestinal inflammation. In addition, TGF-β is a multifunctional cytokine with strong immune suppressive activity, and it includes four different isoforms TGF-β1, TGF-β2, TGF-β3, and TGF-β4 (Kulkarni and Karlsson, 1993; Gorelik and Flavell, 2002; Shiou et al., 2013). The TGF-β1 is one of the key molecules involved in the regulation of the epithelial cell biology and inflammatory response in the intestine (Kulkarni and Karlsson, 1993; Gorelik and Flavell, 2000). The TGF-β1 suppresses inflammation in the absence of mothers against decapentaplegic homolog 7 (*Smad7*) and enhances inflammation in the presence of *Smad7* (Troncone et al., 2018). A recent study showed that when animals were challenged with *Salmonella typhimurium*, *Citrobacter rodentium*, or dextran sodium sulfate to induce intestinal inflammation, the mRNA and protein levels of TGF-β1 and IL-10 were increased in the regulatory subpopulation of innate lymphoid cells in intestine in order to overcome or resolve intestinal inflammation (Wang et al., 2017). Gene expression of *TGF-β4* was increased in the jejunum of broilers with intestinal barrier failure and inflammation induced by *Eimeria* infection and rye-wheat-based diet, suggesting that the increase of *TGF-β4* gene expression is associated with intestinal barrier failure and inflammation (Chen et al., 2015). Considering the lower *NF-κB* gene expression in piglets exposed from maternal MMHAC, the reduction of *TGF-β1* gene expression suggests that piglets from sows fed a diet with MMHAC may have lower inflammatory response than piglets from ITM-supplemented sows. Taken together, maternal supplementation of MMHAC to sows reduced intestinal inflammation and improved intestinal health status in their piglets.

There were, however, no changes in the amounts of inflammatory cytokines (TNF-α, IL-8, and TGF-β) and MUC2 at protein levels in the jejunal mucosa by maternal MMHAC. This does not match to the changes observed at mRNA levels, which makes the interpretation of MMHAC effects complicated. Experimental diets used in this study represent typical commercial diets, including supplements such as phytase, yeast, and choline with antibacterial, anti-inflammatory, and antioxidative functions (Shen et al., 2009; Wiedeman et al., 2018). It is currently speculated that these supplements also affected the production of inflammatory cytokines and MUC2, and thus, the effects of maternal MMHAC shown at the mRNA level were not clearly shown at the protein levels. Previous studies showed that the correlation between gene and protein levels generally is not exactly consistent (Edfors et al., 2016; Liu et al., 2016). Another study also demonstrated that there are variable and complicated processes of RNA transcription, translation, localization, modification, and the regulation of protein degradation (Vogel and Marcotte, 2012). These regulatory processes could affect the relationship between gene transcription by ribosome coding and protein levels (Edfors et al., 2016; Liu et al., 2016).

In conclusion, maternal supplementation of MMHAC to sows during gestation improved intestinal health and skeletal muscle growth in progeny piglets by modulating the expression of mRNA for key regulatory proteins. In the intestine, supplementation of MMHAC reduced inflammation by downregulating *NF-κB* and *TGFβ1* expression. In the *longissimus* muscle, supplementation of MMHAC potentially promoted skeletal muscle growth by upregulating histone H3K9 acetylation, by tendency of downregulating *MRF4* gene expression, and by differentially regulating *DEGS1* gene expression. However, these changes in mRNA expression were not shown at protein levels. Collectively, maternal supplementation of MMHAC could potentially modulate histone acetylation and programming in the fetus during gestation, regulate intestinal health and skeletal muscle development of piglets, and, therefore, lead to enhanced growth of their piglets. However, the results from this study could not directly answer whether the regulation of genes expression measured is mediated by epigenetic modification such as histone acetylation which warrants future investigation.

## Abbreviations

ADFI: average daily feed intake
BW: body weight
DEGS1: delta 4-desaturase sphingolipid 1
DNA: deoxyribonucleic acid
ELISA: enzyme-linked immunosorbent assay
HDAC: histone deacetylases
HEK293: human embryonic kidney 293 cells
IgA: immunoglobulin A
IgG: immunoglobulin G
IgM: immunoglobulin M
IL: interleukin
MEF: myocyte enhancer factor
MHA: methionine hydroxy analog
MHAC: methionine hydroxy analog chelate
MMHAC: mineral methionine hydroxy analog chelate
MRF: myogenic regulatory factor
mRNA: messenger ribonucleic acid
MSTN: myostatin
MTOR: mammalian target of rapamycin
MUC2: mucin-2 protein
MYF: myogenic factor
MYOD: myogenic differentiation
MYOG: myogenin
NF-κB: nuclear factor kappa B
PCR: polymerase chain reaction
RNA: ribonucleic acid
Smad7: mothers against decapentaplegic homolog 7
TGF-β: transforming growth factor-beta
TNF-α: tumor necrosis factor-alpha
VH:CD: villus height to crypt depth ratio
ZNF: Zn-finger proteins
